# Myeloid C-Type Lectin Receptors in Viral Recognition and Antiviral Immunity

**DOI:** 10.3390/v9030059

**Published:** 2017-03-22

**Authors:** João T. Monteiro, Bernd Lepenies

**Affiliations:** University of Veterinary Medicine Hannover, Immunology Unit & Research Center for Emerging Infections and Zoonoses (RIZ), Bünteweg 17, 30559 Hannover, Germany; joao.monteiro@tiho-hannover.de

**Keywords:** C-type lectin receptors, glycans, dendritic cells, macrophages, immunomodulation, antiviral immunity, viral evasion

## Abstract

Recognition of viral glycans by pattern recognition receptors (PRRs) in innate immunity contributes to antiviral immune responses. C-type lectin receptors (CLRs) are PRRs capable of sensing glycans present in viral pathogens to activate antiviral immune responses such as phagocytosis, antigen processing and presentation, and subsequent T cell activation. The ability of CLRs to elicit and shape adaptive immunity plays a critical role in the inhibition of viral spread within the host. However, certain viruses exploit CLRs for viral entry into host cells to avoid immune recognition. To block CLR interactions with viral glycoproteins, antiviral strategies may involve the use of multivalent glycan carrier systems. In this review, we describe the role of CLRs in antiviral immunity and we highlight their dual function in viral clearance and exploitation by viral pathogens.

## 1. Introduction

The immune system is a host defense system composed of cellular and humoral components with the ability to protect the host against pathogens, foreign substances and tissue damage [1]. It can be subdivided into innate immunity [2] and adaptive immunity [3]. Recognition of evolutionarily conserved pathogen-derived ligands, so-called pathogen-associated molecular patterns (PAMPs), by the host innate immune system is mediated by pattern recognition receptors (PRRs). PRRs are essential to initiate innate responses and also contribute to the induction of adaptive immune responses [4,5]. Major classes of PRRs comprise Toll-like receptors (TLRs) [6], nucleotide-binding oligomerization domains (NOD)-like receptors (NLRs) [7], retinoic acid-inducible gene (RIG)-I-like receptors (RLRs) [8], DNA sensors [9], and C-type lectin receptors (CLRs) [10].

Myeloid CLRs in innate immunity are glycan-binding receptors that are specialized in the recognition of glycolipids and glycoproteins present on pathogens and also on host cells [10,11,12]. Since numerous pathogens including bacteria, parasites, fungi, and viruses are coated by glycans, glycan recognition by host CLRs is vital to elicit immune responses. CLRs may impact immunity at several levels, ranging from phagocytosis to the production of effector cytokines and chemokines [13]. Moreover, various CLRs act as endocytic receptors on antigen-presenting cells (APCs), thus are involved in the uptake of pathogens for antigen processing and presentation, and subsequent T cell activation [14].

The role of CLRs in viral recognition is complex. CLRs expressed by APCs, such as dendritic cells (DCs) and macrophages, are able to recognize and quickly respond to a broad range of viruses in order to shape antiviral immune responses. However, some viruses have evolved mechanisms to exploit CLRs to trigger cell entry, to disrupt signaling pathways in the cell, or to inhibit APC effector functions [15].

In this review, we describe the intricate interplay between viruses and CLRs and highlight the role of CLRs in antiviral immunity as well as their exploitation by viral pathogens for immune evasion.

## 2. Viral Glycoproteins—Essential Components for Viral Interactions with Host Cells

Glycosylation is a highly diverse process that produces an abundant and highly regulated repertoire of complex glycans that are covalently attached to proteins and lipids present on the surface of host cells and viruses. In contrast to DNA and protein biosynthesis, the biosynthesis of glycans is not a template-driven process. Instead, it relies on an orchestrated action of glycosyltransferases and glycosidases that are present in the endoplasmic reticulum (ER) and the Golgi network [16,17]. Two major types of glycan structures are linked to proteins: *N*-linked glycans and *O*-linked glycans. *N*-glycosylation is characterized by the attachment of *N*-acetylglucosamine (GlcNAc) and mannose residues to the amide nitrogen of an asparagine residue in the context of the conserved motif Asn-X-Ser/Thr. A complex process of trimming and remodeling of the oligosaccharide by glycosyltransferases and glycosidases, first in the ER and later in the Golgi apparatus, finally leads to the formation of *N*-glycans of the oligomannose type, the complex type, or the hybrid type [18]. All subtypes share a common pentasaccharide core, where the oligomannose type possesses solely mannose residues attached to the core, while the complex type has antennae initiated by GlcNAc attached to the pentasaccharide core, and the hybrid type comprises both mannose and GlcNAc attachments [17,19]. *O-*glycosylation takes place once the protein has transited to the Golgi and carbohydrates are linked covalently via glycosidic linkages from serine or threonine residues. Highly branched glycan structures can be achieved by *O-*glycosylation since each monosaccharide has several hydroxyl groups for linkage and can form glycosidic bonds in an α or β configuration [20].

A common glycan modification at terminal ends of *N-* and *O-*linked glycans, as well as glycolipids, is the attachment of acidic sugars termed sialic acids. Sialic acids are present in the outermost end of glycan chains of all cell surface glycans in higher vertebrates [21]. In humans, the most common sialic acid is α5-*N*-acetylneuraminic acid (Neu5Ac) and derivatives of this nine-carbon backbone carbohydrate into different sialic acid variants can occur by chemical modifications, such as acetylation, methylation and sulfation [21,22]. Sialic acids are negatively charged molecules that are involved in cell adhesion, signaling events to elicit immune responses and key recognized structures by several viruses and bacteria [23].

Viruses as obligatory intracellular pathogens hijack and benefit from the host cell glycosylation machinery [24]. Glycosylation of viral surface proteins takes place upon viral replication inside host cells and it is often essential to maintain the stability of these proteins and viral particles. Moreover, glycan moieties in viral glycoproteins are responsible for a delicate interplay between viruses and host cells through specific interactions with lectins present on the surface of host cells [24,25]. Particularly, *N*-linked high mannose type glycans are often essential for biological functions of viral glycoproteins, such as stability and antigenicity, and it may also impact binding of viruses to host cell receptors and subsequent internalization [26]. Indeed, glycoproteins present in enveloped viruses such as the human immunodeficiency virus 1 (HIV-1), Ebola virus (EBOV), West Nile virus (WNV), Nipah virus, Newcastle virus, and SARS coronavirus (SARS-CoV) were shown to have *N*‑linked glycans that are crucial for protein folding, viral infectivity, and immune evasion (reviewed in [26]). For instance, in hepatitis C virus (HCV), the glycosylation patterns of envelope glycoproteins have a dual role as they contribute to viral entry into host cells on the one hand, and ensure proper protein folding and maturation on the other hand [27]. Consistently, mutagenesis in glycosylation sites of the HCV E1 protein affected translocation of the protein to the viral surface and thus reduced infectivity [28].

Although viral glycosylation is intrinsically connected to the host due to hijacking of the host glycosylation machinery, the glycosylation profiles of viruses and host cells may differ, since viral glycoproteins are often more heavily glycosylated and present different glycan modifications [24]. The gp120 glycoprotein of the HIV-1 envelope represents a meaningful example, as gp 120 comprises more than 20 *N*-linked glycan moieties and is one of the heaviest *N*-glycosylated proteins in nature. In contrast to mammalian glycoproteins that often display complex type *N*-glycans, gp120 predominantly carries oligomannose *N-*glycans due to a hindered processing of glycan intermediates by ER and Golgi network glycoenzymes [29].

The crucial role of viral glycosylation in the biology of infection has prompted research on glycan-binding receptors (so-called lectins) on host immune cells that are capable of recognizing glycan structures on viruses and eliciting immune responses, notably a lectin superfamily termed CLRs.

## 3. Myeloid C-type Lectins Receptors—Pattern Recognition Receptors in Innate Immunity

CLRs are carbohydrate-binding proteins that possess a C-type lectin-like domain (CTLD) [30], a conserved structural motif arranged as two protein loops stabilized by two disulfide bridges at the base of each loop [31]. This key structural motif in CLRs is responsible for the recognition of a variety of ligands, often in a Ca^2+^-dependent fashion through a compact module termed carbohydrate recognition domain (CRD) [12]. Although most CLRs bind to glycoconjugates, they may also recognize non-carbohydrate ligands such as cholesterol [32] and uric acid crystals [33]. Specific amino acid motifs within the CRD confer ligand specificity to CLRs such as the EPN (Glu-Pro-Asn) motif and the QPD (Gln-Pro-Asp) motif that predetermine binding affinity towards mannose/fucose and galactose residues, respectively [30]. The CLR superfamily is the largest and most diverse lectin family in animals and has been classified into 17 groups (I-XVII) based on phylogeny, structural, and functional properties [31]. This review focuses on myeloid CLRs that are predominantly expressed by APCs such as monocytes, macrophages, and DCs. APCs have a pivotal role in pathogen uptake and antigen presentation to naïve T cells on either major histocompatibility complex (MHC)-I or MHC-II molecules [34,35]. Recognition of pathogen-derived glycans by CLRs on APCs contributes critically to these APC functions [36]. In addition, CLR engagement triggers cytokine and chemokine secretion, which is necessary to shape adaptive immune responses [36].

The respective myeloid CLRs belong to group II, V, and VI, according to the above-mentioned classification. Group II is composed of type II transmembrane CLRs, such as the dendritic cell-specific intercellular adhesion molecule-3-grabbing non integrin (DC-SIGN), and the DC‑immunoreceptor (DCIR), which contain a short cytoplasmic tail, a transmembrane domain, an extracellular stalk region and a Ca^2+^-dependent CRD [13,31]. Dependent on the length of the stalk region, different oligomerization states may occur in members of this group. CLRs in group V include, for example, the myeloid DAP-12 associating lectin (MDL-1) and Dectin-1 that possess a short cytoplasmic tail, a transmembrane domain and an extracellular stalk region followed by a single CTLD often lacking typical Ca^2+^ or carbohydrates binding sites [13,31]. Group VI CLRs are type I transmembrane receptors with an extracellular domain encompassing a N-terminal ricin-like domain, a fibronectin type 2 domain and eight to ten CTLDs [13,31]. Prominent CLRs such as the macrophage mannose receptor (MMR) and DEC-205 belong to the latter group.

**Figure 1:**
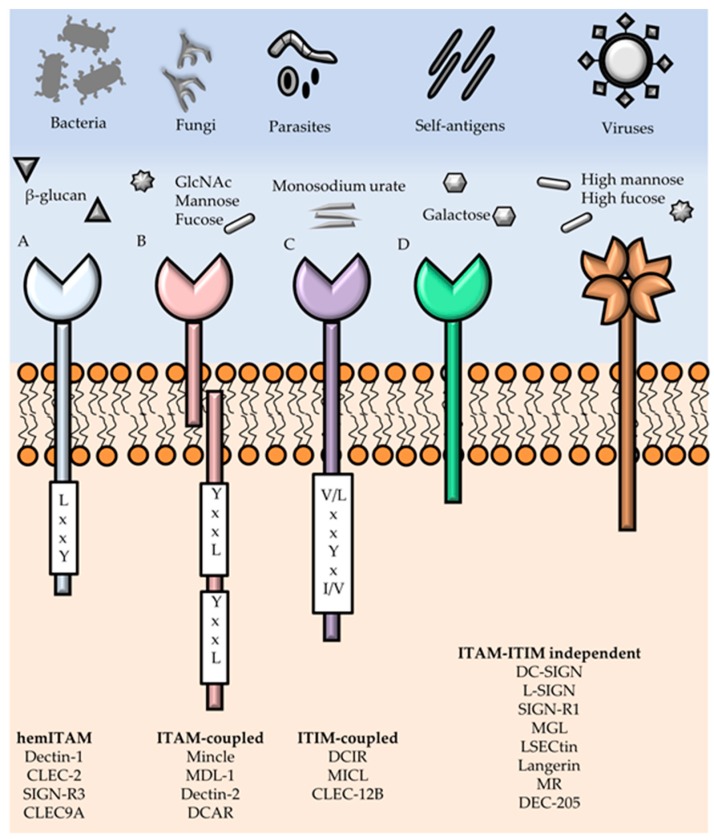
Recognition of pathogen-associated molecular patterns (PAMPs), damage-associated molecular patterns (DAMPs) and signaling motifs of myeloid C-type lectin receptors (CLRs). CLRs expressed by antigen-presenting cells (APCs) are able to recognize PAMPs present on pathogens, including bacteria, viruses, fungi and parasites; and DAMPs in damaged host cells. Recognized ligands cover a vast type of glycan structures, such as fucose, mannose, β-glucan, galactose, GlcNAc, but also non-glycan ligands such as monosodium urate. Upon CLR engagement, a signaling cascade is initiated through binding of early adaptors and the recruitment of kinases or phosphatases. Myeloid CLRs can be subdivided in four distinct groups according to their cytoplasmic signaling motifs and early adaptors: (**A**) hemi-immunoreceptor tyrosine-based activating motif (hemITAM)-coupled; (**B**) ITAM-coupled; (**C**) immunoreceptor tyrosine-based inhibitory motif (ITIM)-coupled; and (**D**) ITAM-ITIM independent CLRs.

Myeloid CLRs may have intracellular signaling motifs and—based on the presence of these motifs—are classified into four main groups: immunoreceptor tyrosine-based activating motif (ITAM)-bearing CLRs, hemi-ITAM (hemITAM)-bearing CLRs, immunoreceptor tyrosine-based inhibitory motif (ITIM)-bearing CLRs, and a group of CLRs lacking typical signaling motifs (Figure 1) [13,37]. ITAMs consist of YxxL/I tandem repeats and are present in ITAM-bearing adaptor proteins such as the FcRγ chain. CLRs such as the DC-associated C-type lectin (Dectin)-2, DC-immunoactivating receptor (DCAR), macrophage inducible C-type lectin (Mincle), and MDL-1 belong to this group of ITAM-bearing CLRs. An intracellular hemITAM motif (a single YxxL/I sequence) is found in CLRs such as Dectin-1, C-type lectin domain family (CLEC)-2, CLEC-9A, and SIGN-R3. Generally, CLRs that carry a hemITAM motif or are associated with ITAM-bearing adaptor proteins activate the spleen tyrosine kinase (SYK) [38]. SYK activation prompts the formation of a ternary complex involving the adaptor proteins caspase recruitment domain-containing protein 9 (CARD9), mucosa-associated lymphoid tissue lymphoma translocation protein 1 (MALT1), and B-cell lymphoma/leukemia 10 (BCL10), which triggers the activation of the transcription factor nuclear factor kappa-light-chain-enhancer of activated B cells (NF-κB) [13,38] and other transcription factors such as the nuclear factor of activated T cells (NFAT) and activator protein 1 (AP-1) [13]. The activation of these signaling cascades in APCs ultimately leads to cytokine production and T cell priming [36]. In contrast, ITIM-bearing CLRs such as the DC immunoreceptor (DCIR), CLEC-12A, and CLEC-12B may inhibit cellular responses through the recruitment of tyrosine phosphatases, such as Src homology region 2 domain-containing phosphatase (SHP)-1 and SHP‑2 [13,39]. The fourth group of CLRs includes members without known ITAMs or ITIMs, such as the MMR, DEC-205, and DC-SIGN that are involved in endocytosis, thus contribute to antigen processing and presentation to T cells [36]. CLR engagement by glycans present in viral glycoproteins often leads to virus internalization into APCs.

**Figure 2:**
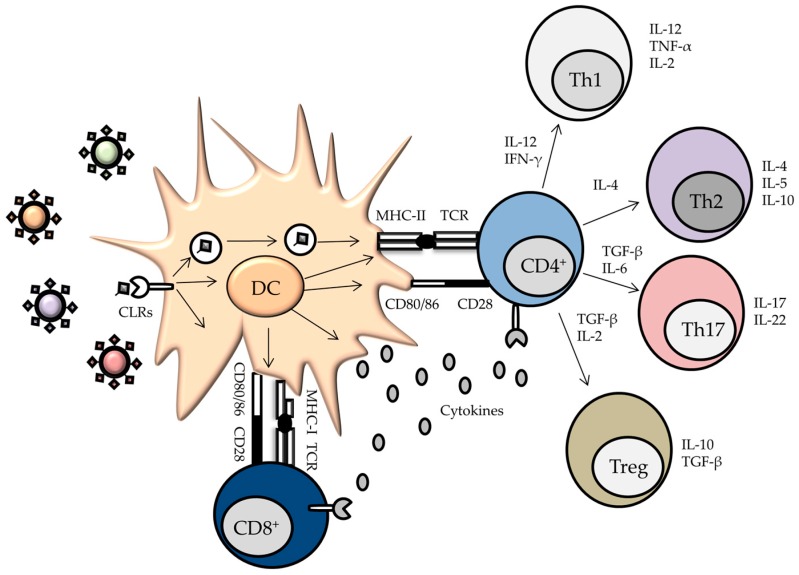
CLRs present on the surface of dendritic cells (DCs) recognize viral antigens to trigger APC activation and subsequent T cell stimulation. Upon binding, endocytosis will take place, resulting in the internalization of the antigens. Further processing in endosomes and lysosomes results in fragmented peptides that are loaded on major histocompatibility complex (MHC) class II and MHC class I molecules for efficient priming of CD4^+^ and CD8^+^ T cells, respectively. Two signals are required for T cell activation by DCs. First, the T-cell receptor (TCR) recognizes MHC/peptide complexes. Second, the costimulatory molecules CD80/CD86 interact with CD28 expressed by the T cell. In addition, DCs express and secrete cytokines. The combination of these signals determines the fate of the activated T cell.

Processing of viral proteins in endosomal compartments finally results in the presentation of antigen-derived peptides on MHC-II molecules to naïve CD4^+^ T cells (Figure 2). Importantly, CLRs are also involved in cross-presentation, thus linking the endocytosis of viral pathogens with the presentation of viral peptides on MHC-I molecules to activate CD8^+^ T cells. In addition, signaling cascades triggered by CLR/virus interactions induce the expression of co-stimulatory molecules and the production of cytokines, therefore contributing to the fate determination of naïve T cells upon activation [40,41]. Thus, CLRs are at the frontline of innate and adaptive antiviral immune responses. In the following sections, the role of selected CLRs in viral recognition and antiviral immune responses will be described.

### 3.1. DC-SIGN

**Figure 3:**
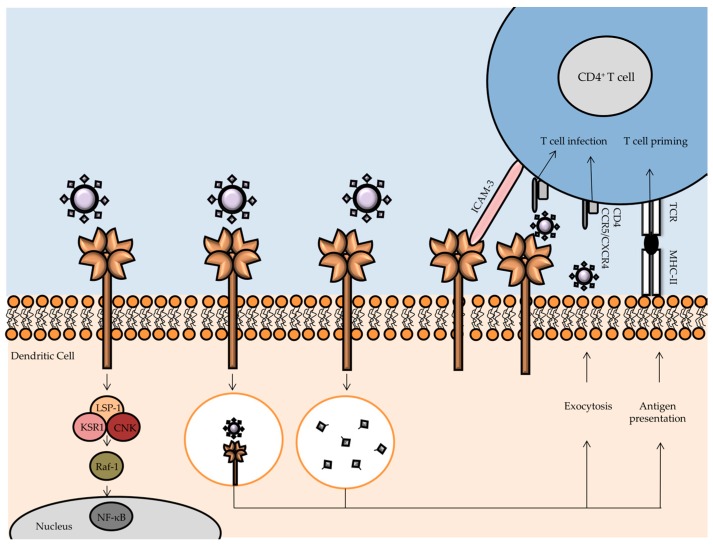
Human immunodeficiency virus type 1 (HIV-1) capture and transmission by DCs. Dendritic cell-specific intercellular adhesion molecule-3-grabbing non integrin (DC-SIGN) present at the surface of DCs neutralizes HIV-1 by virion uptake and signalosome-mediated cytokine production and degradation of virions. Fragmented peptides are loaded on MHC class II molecules for antigen presentation to CD4^+^ T cells in order to prime T cell effector functions and induce an adaptive immune response against the virus. DC-SIGN establishment of a DC-T-cell interaction is accomplished through transient binding to intercellular adhesion molecule (ICAM)-3. However, DC-SIGN is also exploited by HIV-1 for evasion of the immune response by maintenance of intact virions in non-lysosomal endosomes. These virions undergo exocytosis and can infect CD4^+^ T cells in a process called *trans*-infection. *Trans*-infection can also occur without HIV-1 virion internalization. Additional subversion strategies of HIV-1 encompass augmented viral replication via the DC-SIGN signalosome and triggering of DC apoptosis via apoptosis signal-regulating kinase 1 (ASK-1).

Human DC-SIGN, encoded by the CD209 gene, is composed of a short cytoplasmic tail, a transmembrane region, a flexible neck domain involved in oligomerization, and a Ca^2+^-dependent CRD [42,43]. DC-SIGN is mainly expressed by immature or mature DCs, but it is also found in specialized macrophages found in placenta or lung [44]. DC-SIGN contributes to maturation and translocation of DCs to secondary lymphoid organs where antigen presentation to T cells takes place [45]. Interaction between DCs and T cells is facilitated by the interaction of DC-SIGN with intercellular adhesion molecule (ICAM)-3 on the T cell surface [43]. DC-SIGN presents ligand specificity for high mannose glycans and fucose-containing glycans (e.g., Lewis antigens) [43,46]. The neck-repeat domain of DC-SIGN enables the formation of tetramers, thus increasing the binding avidity to glycoconjugates present on pathogens [43]. Numerous enveloped viruses were reported to be recognized by DC-SIGN, including HIV-1, EBOV, HCV, dengue virus (DV), cytomegalovirus (CMV), and SARS-CoV [44,47,48,49]. The level of DC-SIGN expression determines virus capture, indicated by a more efficient HIV-1 binding by DCs (characterized by a high DC-SIGN expression) compared to other cell types expressing DC-SIGN in low abundance [50]. Since glycosylation patterns of viral glycoproteins are governed by the glycosylation machinery of the host cells in which the virus replicates, this strongly affects the DC-SIGN ability to recognize and bind to differentially glycosylated viruses such as HIV-1 and EBOV [51,52]. DC-SIGN mediates capture and further transfer of HIV-1 to T cells (Figure 3) [53,54]. DC-SIGN-mediated HIV-1 infection occurs in DCs that then transport virions from sites of HIV-1 exposure, such as the mucosal membranes or bloodstream, to CD4^+^ T cells in lymphoid tissues [55]. DC-SIGN recognizes high-mannose oligosaccharides on the gp120 molecule of HIV-1, a glycoprotein present in the outer layer of the virus essential for viral entry into host cells [42]. Upon DC-SIGN capture of HIV-1, most of the virions are transported to the proteasome, assisted by the interaction of the cytoskeletal phosphoprotein lymphocyte-specific protein 1 (LSP1), where lysosomal degradation takes place. Further antigen processing and presentation then induces priming of T cells. Moreover, DC-SIGN engagement by HIV-1 activates a signaling cascade that ultimately triggers Raf-1 activation to modulate cytokine responses by NF-κB activation. However, HIV-1 is capable of exploiting the DC-SIGN signaling cascade in order to enhance transcription of the HIV-1 genome by NF-κB-mediated expression [56]. Another exploitation mechanism encompasses the ability of intact DC-SIGN-bound HIV-1 virions of being non-fusogenically internalized into non-lysosomal endosomes, where stability and infectivity of the virions remain intact for several days [57]. It is noteworthy that HIV-1 degradation or persistence inside DCs, as well as DC-mediated virus transmission to CD4^+^ T cells, is strongly dependent on the *N*-glycosylation profile of gp120 [58].

After overcoming degradation in DCs, HIV-1 virions can be transferred to CD4^+^ T cells through the “infectious synapse” in a replication-independent process termed *trans-*infection [59]. In mature DCs, protection of HIV-1 virions from endocytosis occurs to a greater extent compared to immature DCs [60]. HIV-1-bound DC-SIGN also dampens the expression of CD86 and MHC-II molecules, thus affecting antiviral immune responses and facilitating *trans*-infection [61]. In a recent study, it was shown that ligation of DC-SIGN by gp120 sensitizes DCs to undergo accelerated apoptosis in response to a variety of stimuli, mediated by excessive activation of the pro-apoptotic molecule apoptosis signal-regulating kinase 1 (ASK-1) [62].

### 3.2. L-SIGN

Lymph node-specific intercellular adhesion molecule-3-grabbing integrin (L-SIGN, also named DC-SIGNR) is a CLR similar to DC-SIGN, exhibiting a 77% amino acid sequence identity and a similar protein architecture [44]. As DC-SIGN, L-SIGN is a type II transmembrane protein with a short cytoplasmic tail involved in signaling and internalization, a transmembrane region, a neck domain consisting of eight repeat regions of 23 amino acids, and a Ca^2+^-dependent CRD [63]. Oppositely to DC-SIGN, L-SIGN presents a highly variable and polymorphic neck region, which impacts ligand-binding affinity and specificity for viral pathogens [40,44]. The neck region of L-SIGN is essential for tetramerization, thus increasing the binding avidity to multivalent ligands displayed on pathogen surfaces [44]. L-SIGN expression is restricted to endothelial cells present in lymph nodes, placenta, and liver sinusoidal endothelial cells [63]. L-SIGN binds to *N*-linked high-mannose oligosaccharides, with a binding preference for mannosylated residues [44]. In contrast to DC-SIGN, L-SIGN does not bind to blood group antigens [44]. L-SIGN is involved in the recognition and capture of glycan structures present on a variety of pathogens, including bacteria, viruses, fungi and parasites [44]. Similar to DC-SIGN, L-SIGN is also exploited by several viruses, such as HIV-1, EBOV, HCV, hepatitis B virus (HBV), Sindbis virus, SARS-CoV, and Marburg virus (MARV) for glycoprotein-mediated attachment and internalization [64,65,66,67,68,69,70].

Furthermore, WNV, a flavivirus with two viral surface glycoproteins (protein E and prM/M), was shown to bind to L-SIGN with a higher affinity compared to DC-SIGN, resulting in host cell infectivity [71]. Léger et al*.* dissected the differences in the interactions of phleboviruses, including Rift Valley fever virus (RVFV), Toscana virus, and Uukuniemi virus with DC-SIGN and L-SIGN [72]. In this study, an endocytosis-defective mutant of L-SIGN was still capable of facilitating viral infection, highlighting the role of L-SIGN as an attachment factor for phlebobviruses, whereas DC-SIGN acted as an authentic entry receptor [72]. Another study indicated DC-SIGN and L-SIGN as authentic endocytic receptors for influenza A virus (IAV) entry and infection [73]. Sialic acid-deficient Chinese hamster ovary cell lines were resistant to IAV infection, but pH- and dynamin-dependent infectivity was restored by expression of DC-SIGN/L-SIGN. Upon expression of endocytosis-defective DC-SIGN/L-SIGN, IAV was still bound by the cells, but susceptibility to infection was clearly reduced [73].

Collectively, L-SIGN and DC-SIGN were shown to act either as attachment factors for viruses or as authentic entry receptors involved in membrane fusion. Hence, they represent targets for viruses to hijack cellular functions in order to evade immune defenses and spread infection.

### 3.3. LSECtin

Liver and lymph node sinusoidal endothelial cell C-type lectin (LSECtin, or CLEC4G) is located on chromosome 19p13.3 in humans, together with DC-SIGN and L-SIGN [74]. LSECtin shares a 31% and 32% amino acid sequence homology with DC-SIGN and L-SIGN, respectively, and is expressed by liver, lymph node and bone marrow sinusoidal endothelial cells, and by Kupffer cells in the liver [74,75]. LSECtin was reported to recognize glycans containing mannose, *N*-acetylglucosamine (GlcNAc) and fucose residues [75].

LSECtin was discovered to enhance EBOV and SARS-CoV infection, however, in contrast to DC-SIGN and L-SIGN, did not impact HIV-1 and HCV infection [76]. Moreover, viral interactions with LSECtin were not competitively inhibited by the mannose-containing polysaccharide mannan suggesting that this CLR recognizes a more restricted spectrum of viruses presumably in a mannose-independent manner [77]. Similarities between LSECtin and DC-SIGN/L-SIGN in viral recognition range from the expression of this CLR by endothelial cells to the interaction with viral glycoproteins and the subsequent endocytosis of bound ligands [77]. The cytoplasmic domain of LSECtin comprises a tyrosine motif and diglutamic-containing motifs that mediate EBOV internalization through LSECtin. Thus, LSECtin, such as DC-SIGN, might function as a virus uptake receptor [78]. However, in contrast to DC-SIGN/L-SIGN, LSECtin-ligand complexes are not dissociated by a low pH milieu, indicating a differential intracellular trafficking upon endocytosis [77].

LSECtin was described to regulate intrahepatic T cell immunity and to limit liver injury in a model of induced experimental acute hepatitis [79,80]. Using LSECtin-deficient mice in adenovirus infection and HBV replication as murine models for viral hepatitis, Liu et al*.* demonstrated that LSECtin facilitated the reduction of liver inflammation by delaying virus clearance and that this process could be hijacked by viruses as an immune evasion mechanism [81].

### 3.4. Langerin

Langerhans cells (LCs) are a distinct subset of DCs that can be found in the epidermis and oropharyngeal mucosa and are effective APCs capable of inducing T cell responses [82]. LCs express a repertoire of PRRs, including TLRs and CLRs. The CLR langerin is a type II transmembrane protein that has a proline-rich short intracellular cytoplasmic domain and an extracellular domain consisting of a neck-region that forms homo-trimers [83,84]. Although langerin is uniquely expressed by LCs in humans, langerin-positive dermal DCs in mice were also identified [85]. Oligomerization into three identical CRDs homo-trimers), enables to increase ligand avidity and affects the carbohydrate specificity of langerin [84]. The CRD of langerin contains an EPN motif and it was shown that langerin binds to mannose, fucose, and GlcNAc in a Ca^2+^-dependent manner [84]. Notably, interaction of langerin with β-glucan was recently shown, hence diversifying the ligand profile of this CLR [86]. Among other pathogens, langerin recognizes viruses such as HIV‑1 and measles virus (MV) [85,87].

Langerin plays an important role in antiviral responses in immature LCs by capturing HIV-1 for degradation in Birbeck granules [85,88]. In contrast to DC-SIGN that contributes to HIV-1 spread by *trans*-infection of T cells, langerin has a protective role during HIV-1 infection by dampening virus spread [85,88]. HIV-1 infection of LCs occurs when langerin function is impaired or in case of high viral titers. Thus, inhibition of langerin enables HIV-1 infection of LCs and facilitates further virus transmission to T cells [88,89].

MV, a member of the genus *Morbillivirus* in the family *Paramyxoviridae*, is a highly contagious virus that enters the host via the respiratory tract and has a tropism for lymphocytes and myeloid cells. Langerin was shown to be a receptor for MV, although it does not mediate virus entry into host cells directly. Langerin may rather be involved in immunity to MV as both immature and mature LCs efficiently presented MV-derived antigens to CD4^+^ T cells [90]. Recently, langerin was shown to be an authentic receptor for binding and internalization of IAV leading to increased viral infection [91].

### 3.5. Dendritic Cell Immunoreceptor

The DC immunoreceptor (DCIR, also known as C-type lectin superfamily 6, CLECSF6) is a type II transmembrane protein that contains an intracellular ITIM and comprises an unusual sequence in its CRD, where the classical EPN motif is modified by substitution of the asparagine by a serine residue resulting in an EPS (Glu-Pro-Ser) motif [92]. Human DCIR is predominantly expressed by cells of the myeloid lineage, such as DCs, macrophages, neutrophils, and monocytes [93]. The glycan specificity of human DCIR includes fucose- and mannose-containing glycans, as demonstrated by binding to mannotriose and Lewis^b^ [94]. The role of DCIR in immunity is not yet completely understood, but it may be involved in the regulation of DC expansion [95], antigen processing, and antigen presentation for efficient CD4^+^ and CD8^+^ T cell priming [96,97]. Knowledge about DCIR during infections is still at its infancy; however, it has been reported that human DCIR recognizes HIV-1 [98,99,100]. Murine DCIR impacts the immune response during Chikungunya virus (CHIKV) infection [101], but may also be involved in immune pathology during parasite infections, as shown for cerebral malaria [102].

Human DCIR acts as an attachment factor for HIV-1 on DCs and surface expression of DCIR by CD4^+^ T cells is induced by HIV-1 [92,93,98,99]. This increased expression of DCIR by CD4^+^ T cells enhances HIV-1 attachment, viral entry, replication and transfer, finally resulting in a higher virus dissemination [93,99].

CHIKV, an arthropod-borne virus of the *Alphavirus* genus in the family *Togaviridae*, causes rheumatic disease in humans characterized by inflammation and damage of musculoskeletal tissues [101]. Consistent with the role of DCIR as an inhibitory CLR, DCIR^-/-^ mice exhibited more severe disease compared to wild-type control mice following CHIKV infection. Disease exacerbation in DCIR^-/-^ mice included a more rapid and more severe onset of virus-induced edema and enhanced weight loss, increased inflammation and damage in both the fascia of the inoculated foot and the ankle joint, as well as an altered cytokine response [101]. This study suggests a protective role for murine DCIR in limiting CHIKV-induced inflammation.

### 3.6. MDL-1

The myeloid DAP-12-associating lectin (MDL-1, also known as CLEC5A) is a type II transmembrane receptor that is mainly expressed by monocytes, macrophages and neutrophils, but not by monocyte-derived DCs [103,104]. The short cytoplasmic domain of MDL-1 interacts with the ITAM-bearing transmembrane adaptor DAP-12 (12-kDa DNAX-activating protein), leading to phosphorylation and subsequent signaling via the Syk kinase [38,103,105]. MDL-1 does not possess a full set of Ca^2+^-coordinating and carbohydrate-binding sites and exhibits a homodimeric structure at the cell surface [106,107]. Glycan binding specificity still remains unknown [106]. Up to now, MDL‑1 was shown to recognize several viral pathogens, including DV [108], Japanese encephalitis virus (JEV) [109], and influenza viruses [110].

DV, a mosquito-borne virus of the family *Flaviviridae*, is the causative agent of dengue fever. Infections with DV may lead to the development of dengue hemorrhagic fever and dengue shock syndrome [111]. MDL-1 was shown to bind to DV and to activate a signaling cascade accompanied by the release of pro-inflammatory cytokines [108,112] contributing to pathophysiological changes in DV-infected patients [113]. Upon DV recognition by MDL-1, DAP-12 becomes phosphorylated leading to Syk activation. Subsequent induction of the pro-inflammatory cytokines IL-1β and IL-18 and activation of the NLRP3 (NACHT, LRR and PYD domains-containing protein 3 ) inflammasome and caspase-1 trigger cell death (pyroptosis) [114]. Moreover, DV activates nuclear factor (erythroid-derived 2)-like 2 (Nrf2) in murine mononuclear phagocytes to enhance tumor necrosis factor (TNF)-α production through upregulation of MDL-1 [115]. Antibody-mediated MDL-1 blockade attenuated the production of pro-inflammatory cytokines by DV-infected macrophages, suggesting that MDL-1 targeting can be a means to ameliorate tissue damage [108].

MDL-1 is also essential in osteoclastogenesis and bone remodeling upon association with both DAP-12 and DAP-10 in bone marrow-derived osteoclasts [116]. Osteoclasts are multinucleated giant cells that differentiate from macrophages and are involved in bone remodeling [116]. DV infection of osteoclasts was recently shown to upregulate osteolytic activity [117]. Attenuation of DV-induced osteolytic activity was observed in MDL-1^-/-^ mice and, consistently, administration of a MDL-1 antagonist in wild-type mice also inhibited DV-activated osteolytic activity [117].

JEV was shown to bind to MDL-1 and to induce DAP-12 phosphorylation in macrophages as well. Antibody-mediated MDL-1 blockade inhibited JEV-induced pro-inflammatory cytokine release from microglia and prevented bystander damage to neuronal cells. In addition, treatment with an anti-MDL-1 antibody reduced the infiltration of JEV-harboring leukocytes into the central nervous system, attenuated neuroinflammation, and decreased JEV-induced lethality in mice [109]. Thus, MDL-1 plays a critical role in the pathogenesis of Japanese encephalitis and may represent a promising target for the development of new treatments [109].

In a recent study, MDL-1 was demonstrated to interact with the hemagglutinin (HA) protein of influenza viruses [110]. Two modes of interaction can take place between IAV and lectins: calcium-dependent binding of lectins to the viral glycoproteins, and binding of viral HA to sialylated carbohydrates on the lectin (this binding mode mainly occurs in soluble lectins) [25]. Since MDL-1 does not comprise the canonical set of calcium coordinating and carbohydrate binding sites, ligand binding may occur in a Ca^2+^-independent fashion [106]. In addition to identifying MDL-1 as an IAV-recognizing lectin, this study highlighted that antibody-mediated MDL-1 blockade or MDL-1 silencing led to decreased levels of pro-inflammatory cytokines produced by human macrophages. Consistently, MDL-1^-/-^ mice exhibited reduced levels of pro-inflammatory cytokines, decreased immune cell infiltration in the lungs and improved survival compared to wild-type mice indicating a crucial role for MDL-1 in inflammatory responses, thus contributing to influenza pathogenicity [110].

### 3.7. Macrophage Mannose Receptor

The MMR is a CLR expressed by monocyte-derived DCs, dermal DCs, macrophages, and hepatic endothelial cells. It is involved in innate immunity by recognition of glycan structures on pathogens as well in homeostasis by clearance of the pituitary hormones lutropin and thyrotropin [118]. MMR is capable of recognizing mannose, fucose, and GlcNAc residues that are abundant on the envelope glycoproteins of various viruses. MMR comprehends an N-terminal cysteine-rich domain, a fibronectin type II domain, and eight CRDs that bind and release ligands at the low pH environment within endolysosomes in a Ca^2+^-dependent manner. MMR can dimerize; at cell surfaces there is an equilibrium between the monomeric and dimeric form [119]. MMR interacts with a wide range of pathogens, including viruses such as HIV-1, but also binds to fungi, bacteria, and parasites [118]. The MMR is involved in the phagocytosis of pathogens and is internalized into the endosomal system upon ligand binding via clathrin-coated vesicles [120].

MMR was shown to bind to the HIV envelope protein gp120 in its dimeric form via high mannose oligosaccharides [119,121]. MMR plays a substantial role in the binding and transmission of HIV-1 by macrophages as demonstrated by inhibition with mannan, mannose, ethylenediaminetetraacetic acid (EDTA), and soluble mannose-binding lectin [119,121]. Indeed, macrophages were able to mediate transmission of bound HIV to co-cultured T cells, and this transmission could be blocked by inhibitors of MMR binding.

Besides HIV-1, MMR also recognizes envelope glycoproteins of other viruses including the DV envelope (E) glycoprotein. It was shown that MMR bound to mosquito and human cell-produced DV antigen via its CRDs which was abrogated by deglycosylation of the DV envelope glycoprotein [122]. DV infection of human macrophages was efficiently blocked by anti-MR antibodies indicating a functional role for MMR in DV infection. In addition, MMR together with the CLR macrophage galactose-type lectin (MGL; see below) contributes to endocytic uptake of influenza virus into macrophages [123]. MMR bound to influenza virus via its CRDs in a Ca^2+^-dependent manner. Competitive inhibition of MMR using multivalent ligands led to reduced influenza virus infection of macrophages. Interestingly, influenza virus also bound to sialic acid residues present on the MMR suggesting that MMR may act as a secondary receptor or co-receptor for infection of macrophages [123,124]. Another virus recognized by MMR is HBV. MMR was shown to be involved in HBV surface antigen (HBsAg) recognition and uptake by DCs. Since HBsAg-positive DC were frequently found in the liver of HBV patients, MMR-mediated interaction between HBsAg and DCs may occur in the liver as the main site of HBV infection [125].

### 3.8. Macrophage Galactose-Type Lectin

The CLR Macrophage galactose C-type lectin, MGL, is expressed by monocyte-derived immature DCs and macrophages. MGL is a type II transmembrane protein with a ligand specifity for galactose and *N*-acetylgalactosamine (GalNAc) residues [126]. MGL is targeted by viruses for cellular entry and to promote viral dissemination by evasion of host immune responses [126]. EBOV surface glycoprotein (GP) is highly *N-* and *O-*glycosylated and engages in viral attachment and entry into host cells. GP is cleaved into the GP1 and GP2 subunits by furin, where GP1 is mainly associated with interactions with cell surface receptors to mediate viral entry, whereas GP2 facilitates fusion of viral and host membranes at low pH [127]. MGL-expressing cells exhibited a reduced infectivity for pseudo-typed Ebola virus when GP2 of an infectious viral EBOV strain was replaced by GP2 of a non-infectious strain. Hence, MGL seems to promote EBOV infection by recognition of EBOV surface glycoproteins [127,128]. An increased infectivity of MGL-expressing mammalian cells for MARV was also reported, denoting that the heavily glycosylated, mucin-like domain of GP in MARV is required for efficient interaction with this lectin [129]. Additionally, interaction with IAV was also demonstrated, where MGL functions as an attachment and entry receptor for IAV [123,130]. Cells expressing murine MGL1 were infected in the presence or absence of cell surface sialic acids. While cells expressing endocytosis-deficient MGL1 still mediated Ca^2+^-dependent IAV binding, they were less sensitive to IAV infection, suggesting MGL1 as an authentic receptor for both viral attachment and entry of IAV [123,130].

**Figure 4:**
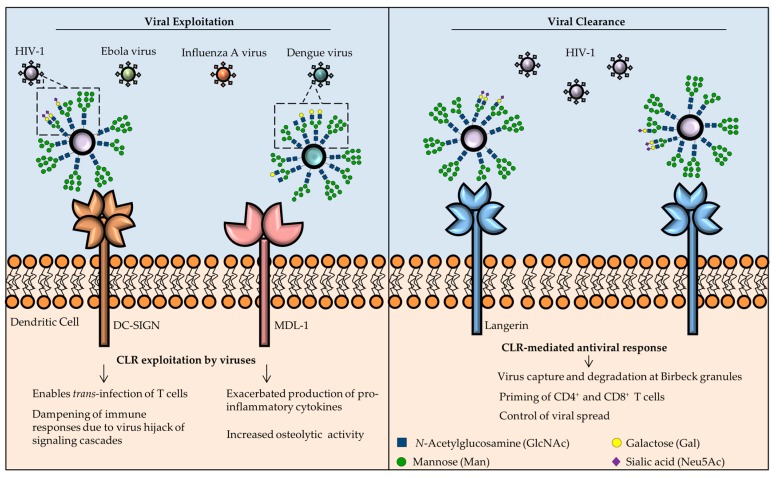
CLRs in antiviral immunity: roles in viral clearance or as targets for viral exploitation. Surface glycoproteins in different enveloped viruses possess glycan moieties (highlighted area), mainly *N*-linked glycans, that are recognized by CLRs and lead to CLR-mediated endocytosis by APCs. Once inside the host cell, viruses may hijack the host’s cellular machinery to ensure viral spreading (left). HIV-1 interaction with DC-SIGN promotes *trans*-infection of T cells and tampers with signaling cascades to promote viral replication or to induce apoptosis of professional APCs to enhance viral infectivity. A different type of exploitation is mediated by viruses, such as DV, through interaction with myeloid DAP-12 associating lectin (MDL-1). In this case, viruses elicit an exacerbated pro-inflammatory response, that damages host cells and increases the susceptibility for viral infection. However, CLRs also have important functions in the clearance of viral infections (right). For instance, langerin captures viruses and directs them towards specialized degradation organelles called Birbeck granules. Langerin is also responsible for priming CD4^+^ and CD8^+^ T cells and to limit viral spread.

In conclusion, CLRs have pleiotropic functions in virus recognition and antiviral immune responses, thus they represent promising targets for new therapies to either block viral entry into host cells or to promote antiviral immune responses (Figure 4). A comprehensive overview of the role of CLRs in viral recognition and immune responses is shown in Table 1.

## 4. Multivalent Glycoconjugates as Antivirals

Since CLRs expressed by APCs represent attractive targets for novel antiviral therapies, glycan-based carrier systems have gained increased attention in the last years, particularly due to their tunability in terms of ligand density, rigidity of the scaffold, and immunogenicity [131,132,133,134]. Since an efficient inhibition of virus/CLR interactions by glycan-based carrier systems requires a high binding avidity, multivalent ligand display using different scaffolds has been employed, including nanoparticles, dendrimers, polymers, fullerenes, and glycan-modified antigens [135,136,137,138,139,140]. Besides the inhibition of viral entry into host cells, multivalent CLR targeting also represents a promising strategy to stimulate APC effector functions to boost immune responses [141,142,143].

DC-SIGN acts as an authentic entry receptor for a diverse range of viruses. As a consequence, targeting strategies towards DC-SIGN using multivalent glycan-based carrier systems have gained a remarkable emphasis [144,145]. The rationale of such strategies is the mimicry of the glycan presentation at the viral surface as the basis for glycan-based antiviral agents. For instance, gold nanoparticles displaying high mannose structures of the HIV undecasaccharide (Man_9_GlcNAc_2_) exhibited affinity towards DC-SIGN in the micro- to nanomolar range [146]. Additionally, DC-SIGN-mediated *trans*-infection of T cells by HIV-1 was substantially inhibited [146,147,148]. Conjugation of glycomimetics to dendrimers has been employed to block viral infection of DC and T cells via DC-SIGN [149,150,151,152]. Glycomimetic compounds inhibited HIV-1 *trans*-infection of T cells by blocking the DC-SIGN/HIV-1 interaction [137,153]. Of note, multivalent glycomimetic display also enabled inhibition of cell infection with EBOV in the nanomolar range in infection assays using pseudotyped EBOV [154].

Glycodendrimers were further used for the preparation of fullerenes or virus-like particles, yielding a wide variety of multivalent glycan architectures [155,156]. Glycodendrofullerenes displayed a low cytotoxicity and allowed for multivalent targeting of CLRs. Competition assays with pseudotyped EBOV particles demonstrated the utility of this strategy, where these compounds presented antiviral activity in the micromolar range [155,156]. Recently, a globular glycofullerene decorated with 120 peripheral glycan subunits, mimicking glycan display on viruses, was shown to be a potent inhibitor of EBOV infection in the subnanomolar range [157]. Thus, multivalent glycan display by fullerenes showed the antiviral potential of this glycan-carrier system as a promising candidate for antiviral strategies by outcompeting viral interactions with lectins on host cells [158]. A multi-layered conjugation approached described by Ribeiro-Viana et al. enabled a high density of glycan display on glycodendrinanoparticles resulting in an efficient inhibition of EBOV infection of DCs and T cells at picomolar concentrations [159].

## 5. Conclusion and Future Perspectives

The interaction of CLRs with viral pathogens is a tightrope walk between pathogen recognition by CLRs for efficient viral clearance and exploitation of CLRs for viral entry into host cells to evade immune responses. CLRs expressed by APCs are able to recognize glycoproteins on the viral surface, and internalize, process and present viral antigens to T cells. Thus, CLRs are often crucial for initiating antiviral immune responses. However, viruses have evolved mechanisms to subvert host defenses, namely by displaying highly glycosylated proteins at their surface that are able to attach to CLRs and promote viral entry into host cells, where hijacking of the host cell biosynthesis machinery permits virus replication and dissemination. Identification of glycans displayed by viruses at their surface is essential to determine glycan recognition by CLRs and to further explore disruption mechanisms of virus-CLR interactions. The glycan array technology provides a robust and reliable platform to identify potential glycan recognition events involved in virus attachment and entry [160]. Structurally defined glycan microarrays as well as shotgun microarrays of natural glycans have paved the way to a more comprehensive knowledge of glycans displayed at the surface of enveloped viruses and glycan recognition by host receptors [160]. Identification of viral glycan structures has enabled the design of glycan-based carrier systems to interfere with viral entry into host cells. Several issues should be addressed in the future, e.g., a comparison of the immunogenicity of the different glycan-based carrier systems, glycan ligand density, spatial orientation, and the impact of multivalency in CLR targeting [133]. Host–virus interactions involve a multiplicity of components that have co-evolved resulting in the ability of viruses to mutate and adapt rapidly in response to selective immune pressures. In an integrated approach, Khatri et al*.* [161] studied the HA glycosylation sites of IAV, often affected by mutations that impair vaccine development, by employing genomics, proteomics, glycomics, molecular modeling and lectin binding data to identify the presence of high mannose glycans at sites implicated in lectin binding. This multidisciplinary approach enabled to elucidate IAV–host interactions and helped to understand the evolutionary selective pressure of the host immune system in virus evolution.

In conclusion, developing new antiviral strategies by exploiting CLRs as targets requires a deeper insight into the role of CLRs in viral recognition and antiviral immunity.

## Figures and Tables

**Table 1:** The role of myeloid C-type lectin receptors (CLRs) in viral infection.

CLR	Location	Ligand Specificity	Function	Virus	Target Protein	Reference
DC-SIGN and L-SIGN	Transmembrane	High mannose High fucose	Enable viral infection	HIV	gp120	[51,54,64]
				EBOV	GP1 subunit	[51,52]
				HBV	HBsAg	[68]
				HCV	Envelope glycoproteins	[65]
				WNV	E protein or prM protein	[71]
				DV	E protein	[48]
				MARV	Envelope glycoproteins	[49]
				SARS-CoV	S protein	[49]
Langerin	Transmembrane	GlcNAc Mannose Fucose	Inhibit viral infection	HIV	gp120	[85,88]
			Enable viral infection	MV	F and H protein	[90]
				IAV	HA	[91]
MMR	Transmembrane	GlcNAc Mannose Fucose		IAV	HA or NA	[123,124]
				HIV	gp120	[119,121]
				DV	E protein	[122]
				HBV	HBsAg	[125]
DCIR	Transmembrane	Mannose Fucose		HIV	gp120	[93,99]
			Inhibit viral infection	CHKV	Envelope glycoproteins	[101]
MDL-1	Transmembrane	Non-defined	Enable viral infection	JEV	Envelope glycoproteins	[109]
				DV	Envelope glycoproteins	[108,112,117]
				IAV	HA	[110]
LSECtin	Transmembrane	GlcNAc Mannose Fucose	Enable viral infection	EBOV	GP	[78]
				HBV	HBsAg	[81]
				SARS-CoV	Envelope glycoproteins	[76]
MGL	Transmembrane	Galactose GalNAc	Enable viral infection	EBOV	GP	[127,128]
				IAV	HA or NA	[123,130]

DC-SIGN: dendritic cell-specific intercellular adhesion molecule-3-grabbing non integrin; L-SIGN: lymph node-specific intercellular adhesion molecule-3-grabbing integrin; MMR: mannose receptor; DCIR: DC immunoreceptor; MDL-1: myeloid DAP-12-associating lectin; LSECtin: liver and lymph node sinusoidal endothelial cell C-type lectin; MGL: macrophage galactose-type lectin; MBL: mannose-binding lectin; SP-A, SP-D: surfactant protein A and D; HIV: human immunodeficiency virus; EBOV: Ebola virus; HBV: hepatitis B virus; HCV: hepatitis C virus; WNV: West Nile virus; DV: dengue virus; MARV: Marburg virus; IAV: influenza A virus; RSV: respiratory syncytial virus; SARS-CoV: severe acute respiratory syndrome coronavirus; JEV: Japanese encephalitis virus; MV: measles virus; CHKV: Chikungunya virus; HA: hemagglutinin; NA: neuraminidase; GPs: surface glycoproteins.
